# Increased risk for dislocation after introduction of the Continuum cup system: lessons learnt from a cohort of 1,381 THRs after 1-year follow-up

**DOI:** 10.1080/17453674.2020.1744981

**Published:** 2020-04-01

**Authors:** Oskari A Pakarinen, Perttu S Neuvonen, Aleksi R P Reito, Antti P Eskelinen

**Affiliations:** Coxa Hospital for Joint Replacement, and Faculty of Medicine and Health Technology, University of Tampere, Tampere, Finland

## Abstract

Background and purpose — The introduction of new total hip replacements (THRs) is known to be associated with an increased risk for complications. On completion of a competitive procurement process, a new uncemented cup system was introduced into general use at our institution in 2016. We launched this study after the introduction to assess (1) the incidence of early dislocations of the old (Pinnacle) and the new (Continuum) cup systems, and (2) whether the cup design would affect the risk for dislocation.

Patients and methods — We assessed the incidence of dislocations after 1,381 primary THRs performed at our institution during 2016. Also, the effect of the cup system (Pinnacle, Continuum with neutral liner, Continuum with elevated rim liner) on dislocation rates was analyzed using a multivariable regression model.

Results — 47 (3.4%) early dislocations were identified. The incidence of dislocations was 1.3% for the Pinnacle, 5.1% for the Continuum with neutral liner, and 1.2% for the Continuum with elevated rim liner. The Continuum with neutral liner was found to have an increased risk for dislocations compared with the Pinnacle (aOR 5, 95% CI 1.4–17). However, when an elevated rim liner was used with the Continuum, the risk for dislocation between the Continuum and the Pinnacle was similar.

Interpretation — Our results emphasize the need for both careful consideration before the introduction of new implants and the systematic monitoring of early outcomes thereafter. The elevated rim liner should be preferred for use with the Continuum cup because of the poor coverage of the neutral liner that may result in dislocations.

Dislocation is one of the most common complications after total hip replacement (THR) with an incidence rate between 0.4% and 4.1% in recent studies (Blom et al. 2008, Itokawa et al. 2013, Ravi et al. 2014, Klasan et al. 2019). Dislocation is often multiple factorial. Many patient-related (e.g., age and BMI), perioperative (e.g., cup malposition), and implant-related (e.g., femoral head size) risk factors for dislocation have been reported (Howie et al. 2012, Danoff et al. 2016, Seagrave et al. 2017). Femoral neck fracture, osteonecrosis, and posterior approach increase the risk for revision due to dislocations (Hailer et al. 2012).

Jumping distance (JD) is defined as the degree of lateral translation of the femoral head center required for the hip to dislocate (Sariali et al. 2009). A smaller JD theoretically increases the risk for dislocation. Femoral head offset, cup inclination and anteversion angles, and femoral head size affect JD (Hamilton et al. 2015). Larger femoral heads decrease the risk for dislocation (Howie et al. 2012), which is partly due to the larger JD (Crowninshield et al. 2004). Nonetheless, a larger femoral head does not guarantee better implant survivorship (Tsikandylakis et al. 2018). The coverage of the acetabular liner is another factor that affects JD. With decreased liner coverage, the JD also decreases.

At our institution, new hip implants were introduced in April 2016. Over the following months, the Pinnacle uncemented porous-coated cup system (DePuy, Warsaw, IN, USA) was gradually replaced by the Continuum, an ultra-porous uncemented cup (Zimmer Biomet, Warsaw, IN, USA). We assessed (1) the dislocation incidences for the different cup systems, and (2) whether the cup design would affect the risk for dislocation even after the confounding factors had been adjusted. Secondarily, we assessed the possible effect of the learning curve associated with the introduction of new hip implants.

## Patients and methods

**Figure 1.**
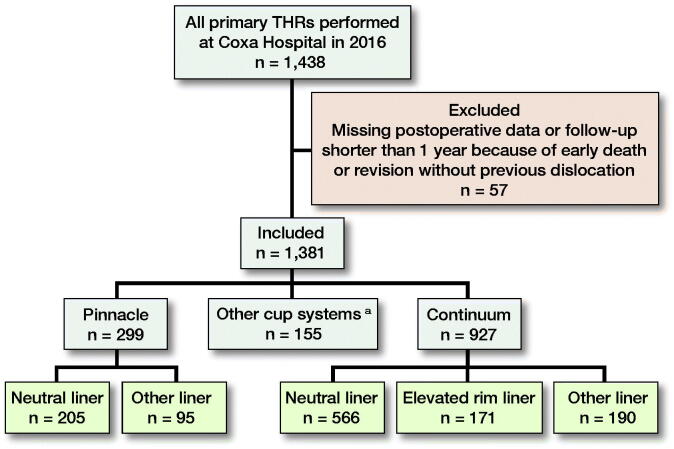
Flow chart of the study population.

Our institution is a high-volume academic tertiary referral center. Currently, more than 1,800 primary THRs are performed annually. In this study, the analyzed data are based on the 1,438 primary THRs performed at our institution between January 1, 2016 and December 31, 2016. Patients who underwent primary THR in both hips during the study period were analyzed as 2 separate operations. The baseline data were obtained from our institution’s electronic health records (EHR) and joint replacement database. 1,347 operations were performed by 30 different surgeons using the posterior approach, while 91 operations were performed using the anterior approach, all performed by the same surgeon. The EHRs of the patients were investigated between December 2017 and August 2018, giving us a minimum of 1-year follow-up (Figure 1).

From our joint replacement database, we collected the following patient demographics and operative details: age, sex, BMI, primary diagnosis, hip range of motion, Charnley classification, surgical approach, femoral head size and model, acetabular cup model, and liner model. From the EHR, we recorded information on all dislocations and revisions that had occurred after primary THR. Those hips that were revised during the first 12 months for reasons other than dislocations were excluded, unless they had dislocated before the revision. We also checked whether these patients had had recurrent dislocations after the first dislocation, and whether the dislocation/dislocations led to revision surgery during the follow-up. In addition, we recorded whether the first dislocation was defined as posterior or anterior in the EHR.

The learning curve effect was assessed by comparing the radiographic results of the acetabular cup positioning of the Pinnacle and Continuum cup systems. Cup malposition is an important risk factor for dislocation (Biedermann et al. 2005). An independent observer (JS) measured cup anteversion and inclination angles from postoperative plain radiographs (AP pelvis) using a previously published technique (Reito et al. 2012). In this study, we defined the “safe zone” for cup position as 10°–25° anteversion and 30°–50° inclination angles, which are considered to be approximately the safest position for the acetabular component (Elkins et al. 2015, Danoff et al. 2016).

### Statistics

**Figure 2.**
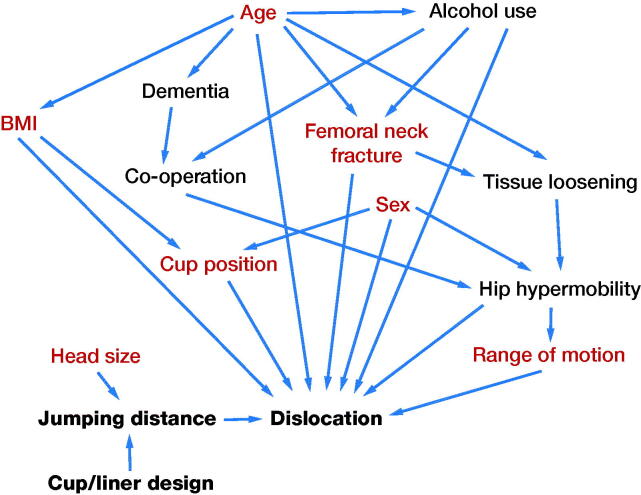
The directed acyclic graph. Factors indicated in red were included in multivariable logistic regression analyses.

95% confidence intervals (CI) for proportions were calculated using the Wilson score interval. Univariable logistic regression was used to estimate the association of baseline risk factors with dislocation. Log-transformed regression coefficients corresponding to unadjusted odds ratio (OR) were reported with CI based on the Wald statistic. Multivariable logistic regression was fitted to estimate the adjusted ORs for dislocation. All potential factors associated with dislocation were included. We used a directed acyclic graph (DAG) in the variable selection to minimize the bias (Figure 2). Based on the DAG, in the final model, ORs for the THR designs were adjusted for age, BMI, diagnosis, sex, cup position, ROM, and head size. 2 different multivariable models were fitted. In the first model, Pinnacle cups were used as a reference with which the Continuum with neutral liner and the Continuum with elevated rim liner were compared. In the second model, we compared the Pinnacle cups with the Continuum cups with neutral liner only. All analyses were performed using IBM SPSS software, version 25 (IBM Corp, Armonk, NY, USA).

### Ethics, funding, and potential conflicts of interest

In accordance with Finnish regulations, informed patient consent was not required as the patients were not contacted. This work was supported by the competitive research funds of Pirkanmaa Hospital District, Tampere, Finland, representing governmental funding. The authors have the following potential conflicts of interest to declare. OP: None. PN: Zimmer Biomet, educational courses. AR: Orion, paid lectures. AE: Zimmer Biomet, paid lectures; Depuy Synthes and Zimmer Biomet, institutional research support (not related to current study).

## Results

The median follow-up was 1.6 years (1.1–2.5). The mean age of patients was 66 years (SD 11) and the mean BMI was 28 (SD 5). 58% of patients were female. Primary osteoarthritis (83%) was the most common reason for operation, followed by femoral neck fracture (5%), osteonecrosis (3%), and developmental dysplasia of the hip (2%). More specific patient characteristics and operative details comparing patients in the Pinnacle and Continuum groups are presented in Table 1.

**Table 1.** Patient demographics and perioperative details. Values are count (%) unless otherwise specified

Factor	Pinnacle	Continuum
Age, mean (SD)	64 (11)	66 (11)
BMI, mean (SD)	29 (4.7)	29 (5.4)
Follow-up, mean years (SD)	2.0 (0.3)	1.5 (0.2)
Female sex	169 (57)	537 (58)
Diagnosis		
Primary osteoarthritis	267 (89)	793 (86)
Femoral neck fracture	6 (2.0)	33 (3.6)
Osteonecrosis	8 (2.7)	25 (2.7)
Developmental dysplasia		
of the hip	9 (3.0)	19 (2.0)
Rheumatoid arthritis	1 (0.3)	17 (1.8)
Other	8 (2.7)	40 (4.3)
Charnley classification		
Unilateral hip OA	132 (62)	445 (64)
Bilateral hip OA	76 (36)	205 (30)
Disability because of		
other diseases	4 (1.9)	42 (6.1)
Head size		
36 mm	221 (77)	750 (81)
32 mm	66 (23)	158 (17)
28 mm	0 (0.0)	4 (0.4)
Cup position, median (range)		
Anteversion	26 (2.6–49)	22 (3.8–53)
Inclination	45 (29–62)	46 (20–71)
Inside safe zone		
Anteversion	116 (39)	651 (71)
Inclination	246 (82)	645 (70)
Both	101 (34)	491 (53)

After 1,381 operations, 47 dislocations occurred (incidence of 3.4%, CI 2.6–4.5). Of the 47 dislocations, 31 were posterior and 11 anterior. The direction of dislocation was unknown in 5 cases. In addition, 20 hips dislocated only once, 20 hips twice, and 11 hips 3 or more times. Over half of the dislocations (24/47) led to revision surgery during the follow-up period. Moreover, 18 of the 24 hips revised for dislocation had had at least 2 dislocations prior to revision. In 4 cases, 2 or more revision surgeries were required.

The incidence of dislocation was 4/299 with the Pinnacle cup system (1.3%, CI 0.5–3.4) and 37/927 with the Continuum cup system (4.0%, CI 2.9–5.5). When the Continuum cup was used with neutral liner, the incidence of dislocation was 29/566 (5.1%, CI 3.6–7.3), while the Continuum combined with elevated rim liner resulted in an incidence of 2/171 (1.2%, CI 0.3–4.2). The Pinnacle used with neutral liner had a dislocation incidence of 3/204 (1.5%, CI 0.5–4.2). In univariable logistic regression analysis (Table 2), the Continuum combined with neutral liner increased the risk for dislocation compared with the Pinnacle. However, the dislocation risk was similar when the Continuum combined with elevated rim liner was compared with the Pinnacle.

**Table 2.** Risk factors for dislocation in univariable logistic regression analysis

Variable	n	Odds ratio (95% CI)	p-value
Cup/liner design			
Pinnacle	299	Reference	
Continuum			
with neutral liner	566	4.0 (1.4–11)	0.01
with elevated rim liner	171	0.9 (0.2–4.8)	0.9
Age (+ 10 years)		1.4 (1.0–1.8)	0.03
Sex			
Male	582	Reference	
Female	799	1.1 (0.6–1.9)	0.8
BMI		1.0 (1.0–1.1)	0.8
Diagnosis			
Primary osteoarthritis	1,149	Reference	
Femoral neck fracture	67	3.9 (1.7–9.3)	0.002
Other	165	1.5 (0.7–3.4)	0.3
Hip mobility			
Internal rotation (+ 5°)		1.3 (1.0–1.5)	0.02
Total ROM (+ 30°)		0.9 (0.8–1.1)	0.3
Femoral head size:			
36 mm	1,061	Reference	
32 mm	248	1.0 (0.4–2.2)	1.0
Approach			
Posterior	1,295	Reference	
Anterior	86	1.5 (0.5–4.1)	0.5
Cup position			
Anteversion 10°–25°	849	Reference	
Anteversion < 10° or > 25°	516	0.8 (0.4–1.4)	0.4
Inclination 30°–50°	1,011	Reference	
Inclination < 30° or > 50°	364	1.2 (0.6–2.2)	0.6

The risk for dislocation using the Continuum with neutral liner compared with the Pinnacle cup remained elevated in the first multivariable analysis. When the Continuum with elevated rim liner was compared with the Pinnacle, however, no difference was observed. In the second multivariable analysis, the neutral liner of the Continuum was compared with the neutral liner of the Pinnacle with the same confounders adjusted. In that analysis, the Continuum’s neutral liner increased the risk for dislocation compared with the Pinnacle’s neutral liner (Table 3).

**Table 3.** Risk for dislocation using the Pinnacle cup system, the Continuum cup system with neutral liner, or the Continuum cup system with elevated rim liner

Cup/liner design	n	aOR (95% CI)	p-value
Pinnacle	299	Reference	
Continuum with neutral liner	566	4.8 (1.4–17)	0.01
Continuum with elevated rim liner	171	1.2 (0.2–7.8)	0.8
Pinnacle with neutral liner	204	Reference	
Continuum with neutral liner	566	5.3 (1.2–24)	0.03

Adjusted for age, sex, BMI, primary diagnosis, total range of motion, femoral head size, cup anteversion angle, and cup inclination angle.

**Figure 3.**
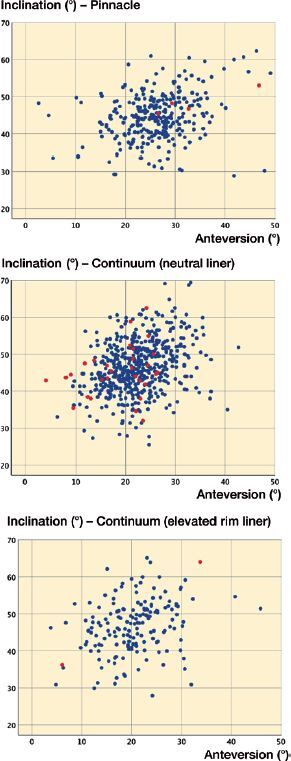
Cup position for the Pinnacle cup (top panel), the Continuum cup with neutral liner (middle panel) and with elevated rim liner (bottom panel). Dislocated hips are marked with red dots.

53% of the Continuum cups and 34% of the Pinnacle cups were positioned inside the safe zone by both anteversion and inclination angles (Table 1). When the Continuum was used with neutral liner, in 17/29 dislocations, the cup was positioned in the safe zone, while neither of the 2 dislocations with the Pinnacle, and none of the 4 dislocations with the Continuum combined with elevated rim liner, occurred inside the safe zone (Figure 3).

## Discussion

In this study, the overall incidence of dislocations at our high-volume academic hospital was 3.4%. Moreover, more than half of the dislocations led to early revision. The incidence of dislocation was unexpectedly high in patients operated on with the Continuum cup and neutral liner (5.1%). Using either the Pinnacle cup (1.3%) or the Continuum cup with an elevated rim liner (1.2%), the dislocation rate was in accordance with our hospital’s quality data over previous years (data not shown). The Continuum cup combined with neutral liner significantly increased the risk for dislocation compared with the Pinnacle cup system, while no statistically significant difference could be detected when the Continuum cup with elevated rim liner was compared with the Pinnacle cup. The Continuum’s neutral liner was also found to increase the risk for dislocation compared with the Pinnacle’s neutral liner.

**Figure 4.**
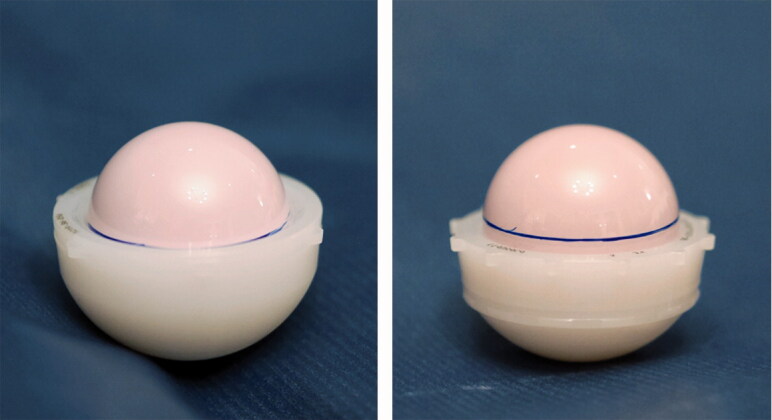
Difference in coverage of the Pinnacle’s and Continuum’s neutral liners. The same ceramic head was first placed in the Pinnacle’s neutral liner (left) and the line at the rim was marked with a pen. Then, the head was moved into the same-sized Continuum’s neutral liner (right).

This study was designed to assess the possible increase in the incidence of dislocation after the change of THR implants at our institution. Before the competitive procurement process, we had mainly used the Pinnacle uncemented porous-coated cup system with neutral polyethylene liner in primary THRs. Starting in April 2016 and over the following few months, the Continuum ultra-porous uncemented cup gradually replaced the Pinnacle as our main cup system. The neutral liner was a natural choice to use with the Continuum because the Pinnacle had been successfully used with neutral liner for many years at our institution. As both cup systems were used as our main system, it is unlikely that the Continuum’s higher dislocation rate was because it had been used in more difficult operations. After the introduction of the Continuum cup as the main cup system, some issues with hip stability were noted in clinical work. Later, it was discovered that the Continuum’s neutral liner had substantially worse coverage compared with the Pinnacle’s neutral liner (Figure 4). The weak coverage of the Continuum neutral liner reduces the JD, and thus explains the high risk for dislocation. The Continuum’s elevated rim liner increases the JD compared with the neutral liner, and therefore decreases the risk for dislocation. These findings led to the launch of the current study.

Previously, trabecular metal cups have been reported to have a higher risk for revision than other uncemented cups (Laaksonen et al. 2018). Since 2011, however, the Continuum cup has also been found to have a higher than anticipated risk for revision in Australian registry data (AOANJRR Annual Report 2011). In the latest AOANJRR annual report, the biggest difference between the Continuum and other total conventional hip prostheses revisions was in the rate of revisions for dislocation; 1.4% of all Continuum cups had had a revision for dislocation, while in other total conventional hips the rate in time-matched comparison was 0.8%. Dislocation was the most common reason for revision with the Continuum, as 38% of all the Continuum revisions were performed due to dislocation compared with 23% for other total conventional hip prostheses (AOANJRR Annual Report 2019). In a recent Finnish register-based study, Continuum cups had a greater risk for revision, and risk for revision because of dislocation compared with other uncemented cups. Moreover, the use of neutral liner with the Continuum cup increased the risk for revision due to dislocation compared with the use of elevated rim liner with the Continuum cup (Hemmila et al. 2019). Our results are clearly in accordance with these recent findings.

As previous studies and register reports indicate, concerns had previously been raised about the reliability of the Continuum cup system, especially regarding the general risk for revision and also the risk for revision due to dislocation. However, other recent studies have concentrated on the risk of revision due to dislocation in register-based settings. To the best of our knowledge, this is the first clinical cohort study that shows an increased risk for dislocations with the Continuum cup system. Moreover, our study is the first to provide causality for the previously observed higher risk for revisions due to dislocations with the Continuum cup, i.e., the poor coverage of its neutral liner that leads to a smaller JD (Figure 4). In our study, however, the risk for dislocation with the Continuum was successfully minimized by using an elevated rim liner that increases the JD. Therefore, we currently prefer to use the elevated liner with the Continuum cup still used at our hospital. We have also learned that when using the Continuum with an elevated rim liner, the anteversion angle should not be exaggerated due to the risk of posterior impingement between the stem neck and the rim of the liner, which can lead to anterior dislocation. In patients who are at high risk for dislocation after THR, we currently favor dual-mobility cups (Harwin et al. 2017).

Although the Continuum cup system was used at our institution on a small scale before the competitive procurement process of 2016, the high dislocation rate could be partially explained by the learning curve. The introduction of new implants in joint replacement surgery has been reported to be associated with a learning curve (Peltola et al. 2013), but there are also less pronounced results (Magill et al. 2016, Mohaddes et al. 2016). On average, the Pinnacle cup was generally positioned at a slightly higher anteversion angle than the Continuum. However, this did not prevent dislocation, as in all dislocations with the Pinnacle the anteversion angle was over 25°. Overall, the Continuum was better positioned than the Pinnacle. As the Continuum has a rougher (macro-textured) surface than the Pinnacle, we have learned that it is better to ream the acetabulum either line-to-line (in most cases), or even perform slight over-reaming (in hard, sclerotic bone) when using the Continuum. With the Pinnacle, on the other hand, we usually under-ream by 1 mm and use line-to-line reaming only in patients with hard, sclerotic acetabular bone. Clearly, the optimal cup position did not provide any protection from dislocation when the Continuum cup was used with neutral liner because most of the dislocations occurred in patients whose cups were positioned inside the safe zone. In comparison, the only 2 dislocations with the Continuum combined with elevated rim liner occurred with very unorthodox cup positions. These results indicate that the high dislocation rates of the Continuum combined with neutral liner cannot be explained by poor cup positioning. However, there may well be other factors that are affected by the learning curve that we are unaware of, so its effect cannot be completely excluded.

In our directed acyclic graph, we did not find any factors that could have affected the choice of cup or liner design. That is because the cup system was changed from the Pinnacle to the Continuum after the competitive procurement process, and at first we mainly used the neutral liner with the Continuum as we had done with the Pinnacle for many years. However, as we observed several dislocations in patients with well-positioned Continuum cups (and neutral liner), we then gradually started to shift from neutral liner to elevated rim liner. At this point, there were still not enough data available to see whether this phenomenon was real or just a chance finding. Thus, the patient characteristics should not have affected the choice of cup system or liner model. Nevertheless, in multivariable regression analysis, we adjusted all the factors that were thought to affect the risk for dislocation and measured them in our data. With or without the adjustment, the difference in dislocation risk (in favor of the Pinnacle) remained obvious. There is always a possibility that shortly after the introduction of the Continuum the elevated rim liner may have been preferred in a small number of patients with slight instability perioperatively. However, if this has caused bias in our material, it is in favor of the neutral liner, not the elevated rim liner.

We acknowledge a few weaknesses in this study. The retrospective study design may enable information bias in the data that we cannot control. Also, adjusting the right confounding factors is not easy and, even when using the DAG, it is just a subjective view. Moreover, we did not have data on the patients’ alcohol use or neurodegenerative disorders, which are known to affect the risk for dislocation (Espehaug et al. 1997, Gausden et al. 2018). Furthermore, because the study cohorts were not randomized, there is always a risk for selection bias. The follow-up period was also rather short, but the majority of dislocations occur within 1 year after surgery (Blom et al. 2008, Meek et al. 2008). In addition, most revisions for dislocation are also performed within 1 year of the primary operation (Hailer et al. 2012). Still, a longer follow-up period would have provided more specific information about the long-term incidence and consequences of dislocations. The strengths of this study are the comprehensive and consistent data from 1 high-volume center that enabled the specific analysis of perioperative factors, such as liners, as there were no differences between hospitals as confounding factors. Indeed, the investigation of the patients’ case records enabled a more exact identification of all the possible dislocations. A clinical cohort study has an obvious advantage over register studies when there is a need to find and to prove causality. The use of DAG also limits the selection bias resulting from a collider (Shrier and Platt 2008).

In conclusion, a marked increase in the incidence of THR dislocations occurred at our high-volume academic joint replacement center after the introduction of a new cup system. This finding can be attributed to the decreased jumping distance and surprisingly low coverage of the neutral polyethylene liners used in the Continuum cup system. Therefore, when a new THR design is brought into general use, the risks for early complications should be considered, and the outcomes carefully monitored, even in high-volume centers with experienced surgeons. There may be design-specific pitfalls in contemporary hip replacement designs that are difficult to tackle if surgeons are unaware of them in advance. We recommend using an elevated rim liner with the Continuum cup system.

OP gathered the data, performed the analyses, and wrote the core of the manuscript. PN co-supervised the project and revised the manuscript. AR coordinated the statistical analyses and revised the manuscript. AE planned the study, revised the manuscript, and supervised the project.

The authors would like to thank Ari Lehtinen for permission to use his photographs in Figure 6, Jaakko Seppänen for measuring the anteversion and inclination angles from the plain radiographs, and Peter Heath for the language editing of the manuscript.
